# The Need for the Closer Monitoring of Novel Drugs in MS: A Siponimod Retrospective Cohort Study (Realhes Study)

**DOI:** 10.3390/jcm12206471

**Published:** 2023-10-11

**Authors:** Arantxa Sancho-López, Belén Ruiz-Antorán, Teresa Iglesias Hernangómez, Almudena Ramírez-García, Irene Gómez-Estévez, Judith Sanabria-Cabrera, Roser Llop Rius, Consuelo Pedrós, Diana Campodonico, Silvia Jiménez-Jorge, Amelia García Luque, Lucienne Costa Frossad França, Eva Montané, Ana Aldea-Perona, Nieves Téllez Lara, Montserrat Bosch Ferrer, Consuelo Rodriguez Jiménez, Elvira Bonilla-Toyos, Julia Sabín Muñoz, Cristina Avendaño-Solá, María Rosario Blasco Quilez, on behalf of the REALHES-Study Group

**Affiliations:** 1 Clinical Pharmacology Department, Hospital Universitario Puerta de Hierro-Majadahonda, Instituto de Investigación Sanitaria Puerta de Hierro-Segovia de Arana, 28222 Majadahonda, Spain; asancho.hpth@gmail.com (A.S.-L.); rgalmu.huphm@gmail.com (A.R.-G.); cavendano.hpth@salud.madrid.org (C.A.-S.); 2 Clinical Pharmacology Department, Hospital Universitario Clínico San Carlos, 28040 Madrid, Spain; tiglesias@salud.madrid.org; 3 Department of Neurology, Hospital Clinico San Carlos, IdISSC, 28040 Madrid, Spain; igomeze@salud.madrid.org; 4 Department of Medicine, Facultad de Medicina, Universidad Complutense de Madrid (UCM), 28040 Madrid, Spain; 5 Clinical Pharmacology Department, Hospital Universitario Virgen de la Victoria, IBIMA_Plataforma BIONAND, Universidad de Málaga, 29071 Malaga, Spain; sanabriajudith@gmail.com (J.S.-C.); ebonillatoyos@gmail.com (E.B.-T.); 6 Platform for Clinical Research and Clinical Trials IBIMA, Plataforma ISCIII de Investigación Clínica, 28029 Madrid, Spain; 7 Clinical Pharmacology Department, Hospital Universitari de Bellvitge, 08907 l’Hospitalet de Llobregat, Spain; rllop@bellvitgehospital.cat; 8 Pharmacology Unit, Department of Pathology and Experimental Therapeutics, School of Medicine and Health Sciences, Barcelona University, 08007 l’Hospitalet de Llobregat, Spain; 9 Unidad de Farmacología Clínica, Consorcio Hospital General Universitario de Valencia, 46014 Valencia, Spain; 10 Clinical Pharmacology Department, Hospital Universitario La Princesa, 28006 Madrid, Spain; diana.campodonico@salud.madrid.org; 11 CTU-HUVR (Clinical Trials Unit-Hospital Universitario Virgen del Rocío), 41013 Sevilla, Spain; silviajimenezjorge@gmail.com; 12 Department of Clinical Pharmacology, Defense Central Hospital, 28047 Madrid, Spain; agarluq@oc.mde.es; 13 Department of Biomedical Sciences (Pharmacology Section), University of Alcalá (IRYCIS), 28801 Madrid, Spain; 14 Department of Neurology, Hospital Universitario Ramón y Cajal, Universidad de Alcalá (IRYCIS), 28801 Madrid, Spain; lucienne.costa@salud.madrid.org; 15 Clinical Pharmacology Department, Hospital Universitario Germans Trias i Pujol, 08916 Barcelona, Spain; emontane.germanstrias@gencat.cat; 16 Department of Pharmacology, Therapeutics and Toxicology, Universitat Autònoma de Barcelona (UAB), 08193 Barcelona, Spain; mb@icf.uab.cat; 17 Clinical Pharmacology Department, Hospital del Mar Barcelona, Clinical Research Unit Research Hospital del Mar Research Institute, Universitat Pompeu Fabra (UPF), 08002 Barcelona, Spain; aaldea@imim.es; 18 Neurology Department, Hospital Clínico Universitario de Valladolid, 47003 Valladolid, Spain; tellezlara@gmail.com; 19 Department of Clinical Pharmacology, Vall d’Hebron Hospital Universitari, Vall d’Hebron Barcelona Hospital Campus, 08035 Barcelona, Spain; 20 Clinical Pharmacology Research Group, Vall d’Hebron Institut de Recerca (VHIR), Vall d’Hebron Hospital Universitari, 08035 Barcelona, Spain; 21 Clinical Trials Unit, Pharmacology Department, Complejo Hospitalario Universitario e Canarias, 38320 Santa Cruz de Tenerife, Spain; conrodjim@gmail.com; 22 Neurology Department, Hospital Universitario Puerta de Hierro-Majadahonda, Instituto de Investigación Sanitaria Puerta de Hierro-Segovia de Arana, 28222 Majadahonda, Spain; julia.sabin@salud.madrid.org (J.S.M.); charoblascoquilez@yahoo.es (M.R.B.Q.)

**Keywords:** multiple sclerosis, siponimod, pharmacovigilance, lymphopenia, real-world evidence

## Abstract

Background: Severe cases of lymphopenia have been reported during siponimod clinical trials, which may negatively impact its benefit/risk profile. Objective: We aimed to evaluate the incidence of lymphopenia following the initiation of siponimod treatment in clinical practice. The secondary objectives included the analysis of factors predisposing to and the clinical relevance of lymphopenia events. Methods: In this multicenter retrospective cohort study, information collected from the medical records of 129 patients with MS from 15 tertiary hospitals in Spain who initiated treatment with Siponimod were followed-up for at least 3 months, including at least one lymphocyte count evaluation per patient. Results: Of the 129 patients, 121 (93.6%) reported lymphopenia events, including 110 (85.3%) with grade ≤ 3 and 11 (8.5%) with grade 4 lymphopenia, higher than those reported in the pivotal clinical trial (73.3% and 3.3% for grade ≤ 3 and grade 4 lymphopenia, respectively). The study included an unexpectedly high proportion of male subjects (72.9%), which might have led to an underestimation of the actual magnitude of the risk. Conclusions: In this study, the incidence and severity of lymphopenia after starting siponimod treatment were higher than those reported in previous clinical trials. Therefore, our results reinforce the need for the closer monitoring of novel MS drugs in clinical practice, as well as larger and longer follow-up studies to properly characterize this risk.

## 1. Introduction

Siponimod (Mayzent^®^) was recently launched in the Spanish market for the treatment of patients with secondary progressive multiple sclerosis (SPMS) presenting an active disease [1].

Siponimod is a sphingosine-1-phosphate (S1P) receptor modulator that acts as a functional antagonist for S1P1 receptors on lymphocytes, preventing egress from lymph nodes. This reduces the recirculation of T cells into the central nervous system (CNS) and limits central inflammation. This redistribution of lymphocytes to secondary lymphoid tissues induces a dose-dependent reduction in the peripheral blood lymphocyte count to 20–30% of the baseline value, according to the confirmatory trials submitted for filing in the European Union (EU). Lymphocyte counts usually return to the normal range within 10 days of ceasing therapy in most patients, but residual lowering effects may persist for up to 3–4 weeks after the last dose [2].

This has led EU regulators to include a recommendation in the Summary of Product Characteristics (SmPC) for monitoring the peripheral lymphocyte count before the start of treatment and periodically afterwards, with dose-adjustment requirements (i.e., a reduction to 1 mg (if taking a 2 mg dose) or the withdrawal of treatment) in the case of grade 4 (<200/mm^3^) lymphopenia. However, no recommendations exist for grade 3 (i.e., <300/mm^3^) sustained (more than 6 months) lymphopenia, despite the theoretical risk for severe infections, whilst the need for treatment interruptions to manage lymphopenic events are not exempt from a risk of relapses for the subjects [3,4,5].

On the other hand, although the association of lymphopenia with medicinal products that block the S1P receptor, like fingolimod or siponimod, is well known, uncertainties regarding the factors accounting for the high inter- and intraindividual variability observed in the frequency and magnitude of lymphopenia still remain [6,7,8,9]. Although grade 4 lymphopenia was reported in 3.3% of patients treated with 2 mg siponimod in the previous double-blind trials [1], we hypothesized that higher rates of severe lymphopenia would occur in clinical practice given the restrictive selection criteria applied in the clinical trials [1], which might have led to an underestimation of the actual magnitude of the risk. The high selectivity of the study population also made it difficult to identify potential predisposing risk factors based on age or prior treatments. Case reports of a higher intensity or duration of lymphopenia compared to those in clinical trials have already been reported [10].

The generation of evidence for the effectiveness and safety of newly marketed medicinal products based on their use in healthcare practice, outside the controlled environment of clinical trials, is critical. Siponimod is subject to additional monitoring, which will expedite the detection of potential new information about its safety. In this context, a retrospective observational study was proposed to evaluate the real incidence of lymphopenia during the first three months after starting treatment with siponimod in real-life patients. A secondary objective was to analyze the baseline characteristics that could potentially predispose one to lymphopenia, either by modifying the pharmacokinetic (age, sex, CY2PC9 genotype, weight, dosage, or duration of treatment) or pharmacodynamic (previous and concomitant therapies received) properties of siponimod. Finally, the risk of infectious adverse events (AEs) associated with lymphopenia events was analyzed. 

## 2. Materials and Methods

This was a multicenter retrospective cohort study performed in 15 tertiary hospitals of five Regional Health Services in Spain. The study recorded data from all adult patients with SPMS treated with siponimod in any of the participant centers since its launch into the Spanish market in April 2021 until September 2022.

Patients with no follow-up for at least 3 months or the complete absence of a lymphocyte count evaluation were excluded. No other inclusion or exclusion criteria were specified. 

Demographic and clinical baseline characteristics and siponimod-treatment-related, laboratory, and safety data were extracted from the medical records using a standardized data collection electronic form. Patients were followed-up according to clinical practice at each study center. Data were collected retrospectively and included all available information from the start of treatment up to a maximum of 18 months after treatment initiation. Data were collated by a primary reviewer at each study center and subsequently checked for completeness and inconsistencies by a senior physician.

The primary outcome of the study was to determine the incidence of lymphopenia after initiating siponimod treatment in patients with SPMS in the clinical practice setting. The secondary outcomes included the degree and severity of lymphopenia AEs, according to the National Cancer Institute Common Terminology Criteria for AEs (NCI-CTCAE); the incidence of infectious AEs; potential risk factors for the development of lymphopenia; and siponimod’s overall safety profile. 

All statistical analyses were conducted using SPSS v.21 (IBM^®^ Corporation, Somers, NY, EE.UU.) and Epidat 3.1 software. A two-sided type I error of 5% was applied in all statistical analyses. For the descriptive analyses, categorical variables were described with frequencies and percentages, and mean (standard deviation, SD) and median (interquartile range (IQR): 25th–75th percentiles) were used for continuous variables. For the inferential analyses, a multivariable logistic regression model was used to identify predictive factors of lymphopenia and severe lymphopenia events. A range of continuous and categorical variables were tested in the model (Table S1). For each tested covariate, a univariate model was estimated. Covariates with *p* < 0.05 for likelihood ratio testing in the univariate analysis were included in a multivariate model, and the selection of independent covariates was based on a backward elimination procedure, retaining covariates with *p* < 0.05. 

This study was approved by the Research Ethics Committee of Hospital Universitario Puerta de Hierro, Madrid, Spain (approval number 148/21), which waived the need for patient informed consent. The study complied with the provisions in EU and Spanish legislation on data protection and the Declaration of Helsinki 2013. The study was registered on 17 January 2022 with the European Network of Centres for Pharmacoepidemiology and Pharmacovigilance (ENCePP) (EUPAS45187). 

## 3. Results

A total of 129 patients with SPMS treated with siponimod were included in this study. The demographic and clinical characteristics of the study cohort are reported in Table 1. The median patient follow-up time was 131 days (IQR 72–190 days).

The proportion of male patients was 72.9%, the mean age at diagnosis was 35.3 years (SD 10.9), and the mean age at the start of siponimod treatment was 53.2 years (SD 9.5). The baseline CYP2C9 genotype was available for 84.5% (109/129) of patients and was the *1*1 genotype in 46.5% of patients, *1*2 in 24.0% of patients, *1*3 in 11.6% of patients, and *2*2 in of 2.3% patients. The most common MS therapies prior to siponimod were fingolimod (15.5%), dimethyl fumarate (DMF) (12.4%), and rituximab (12.4%), followed by glatiramer and teriflunomide (8.5% of patients each), whilst 13.2% of patients were MS treatment-naive. The median washout time from prior MS therapy in our study was 2.2 months. A total of 18 out of 129 (13.9%) patients had some degree of lymphopenia at baseline, with 6 (4.7%) subjects having grade 1 lymphopenia, grade 2 lymphopenia, and grade 3 lymphopenia, respectively. A total of 18 subjects were on an initial maintenance dose of 1 mg, which was in 15 out of the 18 cases guided by the presence of genotype *1*3. 

In our study, the mean (SD) number of CBCs post-treatment was 2.7 (1.9), and the median (IQR) was 1 (1–3). A total of 46 (35.7%) subjects had just one CBC, 31 (24%) had two CBCs, and 52 (40.3%) had three or more CBCs. One hundred and twenty-one out of one hundred and twenty-nine (93.8%) patients reported lymphopenia during the follow-up. Grade ≤ 3 lymphopenia was reported in 85.3% (110/129) of patients, and grade 4 in 8.5% (11/129) of patients (Table 2). The mean percentage reduction in peripheral lymphocyte count was 61.0% (30.1%), i.e., siponimod caused a reduction in peripheral lymphocyte count to 39% of the baseline value.

**Figure 1:**
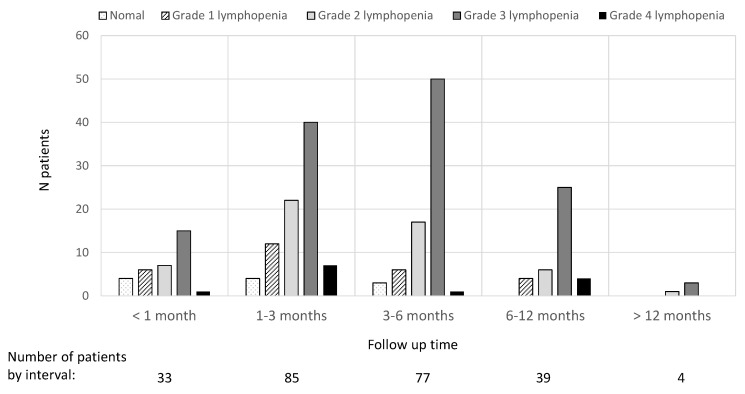
Lymphocyte count results at different times elapsed after treatment initiation.

Among the 129 patients, the timing of first monitoring was highly variable, i.e., within the first month for 26% of patients, 1–3 months for 52% of patients, 3–6 months for 20% of patients and beyond 6 months for 2%. The results for the presence of lymphopenia and its severity at each of the established timepoints after treatment initiation are shown in Figure 1. 

**Figure 2:**
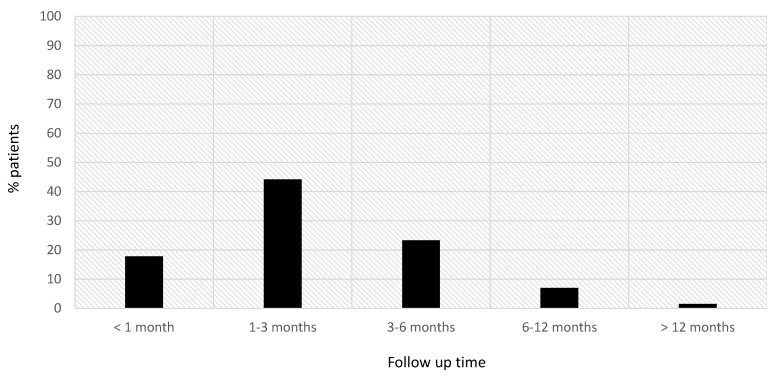
Time at which the maximum grade of lymphopenia was detected in our sample.

The maximum grade of lymphopenia was reported within the first 3 months after starting siponimod treatment for 66.1% (80/121) of patients and within the first 6 months for 98.3% (119/121) of patients (Figure 2).

According to the results of the multivariate analysis, factors associated with grade 3–4 lymphopenia were female sex, lymphocyte values at baseline (mean (SD) 1.40 × 10^9^/L (0.67) vs. 1.92 × 10^9^/L (1,29)), and prior treatment with DMF. The presence of CYP2C9 genotype *2*2 and prior treatment with DMF were found to be significantly associated with grade 4 lymphopenia (Table 3 and Table S2).

**Figure 3:**
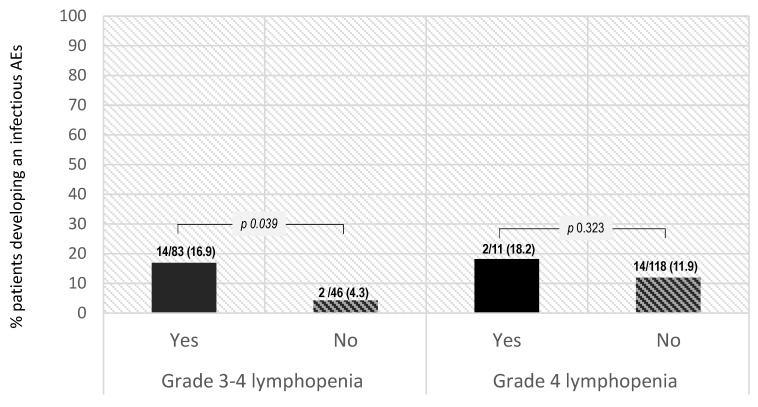
Probability of developing an infectious AE and development of lymphopenia.

The clinical relevance of siponimod-induced lymphopenia events was determined by the incidence of infectious AEs. In our study, the probability of developing an infectious AE was significantly higher in patients who developed grade 3–4 lymphopenia vs. those with lower degrees of lymphopenia or no-lymphopenia (16.9% vs. 4.3%, OR 4.46 (95% confidence interval (CI) 1.97–20.59, *p* value 0.039)). Higher rates of infectious AEs were also reported by patients with grade 4 lymphopenia alone (18.2% vs. 11.9%, OR 1.65 (95% CI 0.32–6.43, *p* value 0.323)); however, the differences were not statistically significant due to the small sample size in this subgroup (Figure 3).

The overall incidence of AEs was 46.5% (Table 4). Serious AEs (SAEs) were reported by 6.2% (8/129) of patients, including seven cases of lymphopenia and one subject with hepatotoxicity. None of the subjects who required an initial dose adjustment due to the presence of genotype *1*3 developed grade 4 lymphopenia. The incidence of infectious AEs was 12.4% (16/129) and included a total of 18 AEs in 16 patients (seven COVID infections, five non-specified infections, four urinary tract infections, and two conjunctivitis cases). Dose adjustments due to AEs were required for 16 patients (12.4%), 15 due to lymphopenia and 1 due to COVID infection. Out of the 15 subjects with lymphopenia, five had grade 4, eight had grade 3, and two had grade 2 lymphopenia. Increments in liver transaminases were frequently reported, in 14.0% (18/119) of patients treated with Siponimod, and 30.3% (39/119) of patients showed increased levels of alanine transaminase (ALT) and aspartate transaminase (AST) during the follow-up period.

## 4. Discussion

This study evaluated the risk of lymphopenia induced by siponimod treatment initiation in patients with MS. Lymphopenia of any grade was observed in 93.6% of the patients included, comprising 110 (85.3%) with grade ≤3, 11 (8.5%) with grade 4, and none with grade 5 lymphopenia. Lymphopenia events were deemed clinically relevant in a substantial proportion of patients considering the incidence of infectious AEs (12.4%) and dose adjustments due to AEs (required in 16 patients (12.4%), including 15 patients due to lymphopenia). The probability of developing an infectious AE was significantly higher in patients who developed grade 3–4 lymphopenia (70.1% subjects) vs. those with lower degrees of lymphopenia or no lymphopenia: 16.9% vs. 4.3%, OR 4.46 (95% CI 1.97–20.59, *p* value 0.039). In addition, numerically higher rates of infectious AEs were also observed in patients with grade 4 lymphopenia (18.2% vs. 11.9%, OR 1.65 (95% CI 0.32–6.43, *p* value 0.323)). 

Notably, in our study, the mean percentage reduction in peripheral lymphocyte count was 61.0% of the baseline value, slightly lower than that reported in previous clinical studies (70–80%) [1]. However, substantial variability was noted, with a non-negligible proportion of patients showing higher than expected mean percentage reductions. The incidence of severe grade 4 lymphopenia was substantially higher than that reported for siponimod in the pivotal clinical trial (EXPAND Study), with an incidence of lymphopenia in 1% of patients (severity not reported) [9], and even higher compared to that reported for a 2 mg dose in the controlled studies (3.3% of patients) [1]. In this study, female sex, lymphocyte values at baseline, and prior treatment with DMF were found to be associated with grade 3–4 lymphopenia. On the other hand, prior treatment with DMF and CYP2C9 genotype *2*2 were significantly associated with grade 4 lymphopenia. 

We found some differences in the baseline characteristics of our study population compared to those of the population included in the EXPAND Study, which may have accounted for the discrepant results regarding the incidence/severity of lymphopenia. Strict exclusion criteria were applied in the EXPAND Study with respect to washout from prior MS treatment that are rarely applied in clinical practice due to the risk of flares. Our study included a more heavily pretreated population, given the lower proportion of MS treatment-naive patients (13.2% vs. 21.7%) and the substantially higher portion of patients previously exposed to DMF, teriflunomide, ocrelizumab, rituximab, alemtuzumab, mitoxantrone, or natalizumab, while this information was anecdotal in the EXPAND Study [9]. Despite the higher proportion of patients previously treated with MS therapies that required a washout time of at least 2 months, 6 months, or even 1 or 2 years, the mean (SD) washout time from prior MS therapy in our study was 49.8 (113.0) days. Thus, a residual effect from prior therapy could not be excluded. Indeed, 18 out of 124 (17.8%) subjects had some degree of lymphopenia at baseline in our study, while these patients were excluded from participation in the EXPAND trial. 

Despite the EU regulatory requirements established in the SmPC, in our study, a high proportion of patients were not genotyped before starting siponimod treatment (15.5%), which might partially explain the increased risk observed. In addition, in our study, a total of 31 (24%) and 3 (2.3%) subjects had the CYP2C9*1/*2 and CYP2C9*2/*2 genotypes, respectively. The absence of recommendations in the siponimod drug label for CYP2C9*2/*2 and *1/*2 individuals does not mean that they are not at risk for adverse drug reactions but indicates an inadequate evaluation in premarketing studies. Indeed, published evidence supports the inference of a CYP2C9 intermediate metabolizer (IM) phenotype with an activity score (AS) of 1 for patients with two reduced-function alleles (e.g., *2/*2) or one normal-function allele plus one non-functional allele (e.g., *1/*3) [10,11,12]. Hence, the dosing recommendation may be the same for CYP2C9*2/*2 and *1/*3 patients. In addition, CYP2C9 activity might also be reduced in CYP2C9*1/*2 subjects [10]. Since the CYP2C9 activity is reduced in CYP2C9 *2/*2 and *1/*2 individuals, to avoid the risk of ADRs, some researchers have suggested that CYP2C9*1/*2 individuals might require a 25% dose reduction and CYP2C9*2/*2 individuals a 50% dose reduction, the same as patients with a CYP2C9 *1/*3 genotype [10].

Our results indicated there might be other factors which would predispose some patients to develop severe lymphopenia, beyond a homozygous and heterozygous CYP2C9 *3 genotype. Among them, prior MS therapies and an insufficient washout time; characteristics like gender and weight, which were identified as potentially influencing changes in lymphocyte count and recovery time; and other CYP2C9 reduced-activity phenotypes require further investigation. Until further data are available to confirm these findings, patients with SPMS should be closely monitored. 

This study included all patients who had initiated siponimod therapy in any of the 15 participating centers from five different regions in Spain [2]. Thus, this can be considered a representative sample of Spanish clinical practice. However, our study sample was selected within a limited period of time, right after siponimod was commercially available on the Spanish market, which might explain the higher-than-expected proportion of male subjects in our study. Since males tend to experience a more aggressive course of MS, this might just reflect the subset of SMPS subjects in whom treatment was prioritized based on the medical need to access a potentially effective treatment and not the actual target population for siponimod in broad terms. Given this, based on the available evidence for siponimod that the presence of more than one of these factors, e.g., female gender and low weight, might have a substantial effect on lymphocytes, the inclusion of a high proportion of males in our study might have led us to underestimate the actual magnitude of the risk.

Our findings are consistent with reports across Europe of higher rates of lymphopenia and discontinuations due to AEs in patients initially prescribed siponimod [13,14]. This has raised concerns given the implications for clinical management, including the increased risk of relapses associated with dose reductions and dose discontinuations. 

Our study had some limitations. This was a small retrospective cohort study, and information was collected from medical records based on the actual routine practice in each center. This explains why some subjects started siponimod treatment without a CYP2C9 genotype at baseline, the potentially insufficient washout time from prior therapies and the fact that treatment was started despite the presence of some degree of lymphopenia in some patients, and the high variability in the timing of the complete blood count (CBC) analyses. In addition, the higher-than-expected proportion of male subjects might have led us to underestimate the actual magnitude of the risk in the target SPMS population, where a higher proportion of females would be treated. Furthermore, the study objectives focused on the incidence and clinical relevance of lymphopenia events in the short term, whereas no information on efficacy or long-term safety was collected. Finally, information about the need for dose reductions and/or treatment interruptions/discontinuations due to relevant lymphopenia was not systematically collected; therefore, the actual clinical relevance of lymphopenia events was likely underestimated, given the clinical implications of these decisions.

The high incidence and severity of lymphopenia events observed in our study raise concerns regarding the treatment management of MS patients in clinical practice. These results point out the need for larger studies to better characterize the risk of lymphopenia events and identify possible associated risk factors. It is important to better understand if an insufficient washout period or residual lymphopenia from prior therapies, a measure that is difficult to appropriately follow-up in clinical practice due to the risk of flares, would explain the observed differences or if other individual characteristics, including sex or other genetic factors, are involved. There is a need to better understand the clinical consequences of this phenomenon in the current clinical landscape; if it poses an increased risk for severe infections and/or a worse response to vaccination, as has been reported for anti-CD20 therapies and COVID19 disease/vaccines; and to what extent prior therapies frequently used in clinical practice could influence it [15,16]. Until further information is available, it is necessary to reinforce the current SmPC recommendations for closer CBC monitoring soon after starting siponimod treatment in patients with MS.

## 5. Conclusions

In our study, the incidence and severity of lymphopenia after starting treatment with siponimod were higher than those reported in previous clinical trials. Our results reinforce the need for the closer monitoring of novel drugs for MS in clinical practice, as well as for larger and longer follow-up studies to properly characterize this risk.

## Figures and Tables

**Table 1:** Clinical and demographic characteristics of the enrolled cohort.

	Overall (N = 129)
Gender, male, N (%)	94 (72.9)
Age at MS diagnosis (years), mean (SD)	35.3 (10.9)
Age at start of siponimod treatment, mean (SD)	53.2 (9.5)
Body mass index (BMI), mean (SD)	23.5 (4.3)
Comorbidities, N (%)	
Hypertension	18 (14.0)
Autoimmune disorders	11 (8.5)
Cardiovascular disease	7 (5.4)
Respiratory disease	3 (2.3)
Liver disease	0 (0.0)
Renal impairment	0 (0.0)
CYP2C9 genotype, N (%)	
*1/*1	60 (46.5)
*1/*2	31 (24.0)
*2/*2	3 (2.3)
*1/*3	15 (11.6)
*2/*3	0 (0.0)
*3/*3	0 (0.0)
Other	0 (0.0)
Unknown/not determined	20 (15.5)
Prior MS treatment before starting siponimod, N (%)	
Fingolimod	20 (15.5)
Rituximab	16 (12.4)
Dimethyl fumarate	16 (12.4)
Teriflunomide	11 (8.5)
Glatiramer acetate	11 (8.5)
Interferon beta 1a	9 (7.0)
Ocrelizumab	8 (6.2)
Interferon beta 1b	6 (4.7)
Natalizumab	6 (4.7)
Alemtuzumab	4 (3.1)
Cladribine	1 (0.8)
Mitoxantrone	0 (0.0)
Ofatumumab	0 (0.0)
Ozanimod	0 (0.0)
Posenimod	0 (0.0)
Time since discontinuation of prior treatment until the start of siponimod (months), median (IQR)	2.2 (0.23–15.6)

SD: standard deviation; BMI: body mass index; MS: multiple sclerosis; IQR: interquartile range.

**Table 2:** Lymphopenia laboratory test outcomes.

N (%) Patients	Overall (N = 129)
Lymphopenia during follow-up *	
No lymphopenia	8 (6.2)
Grade I (<LLN–800/mm^3^)	25 (19.4)
Grade II (<800–500/mm^3^)	39 (30.3)
Grade III (<500–200/mm^3^)	79 (61.2)
Grade IV (<200/mm^3^)	11 (8.5)
Grade V (death)	0 (0.0)
**Maximum lymphopenia during follow-up**	
No lymphopenia	8 (6.2)
Grade I	15 (11.6)
Grade II	23 (17.8)
Grade III	72 (55.8)
Grade IV	11 (8.5)
Grade V	0 (0.0)

LLN: lower limit of normality. * Number of patients who presented alterations in the CBCs performed during their clinical follow-up. Patients may have undergone more than one complete blood count during the follow-up.

**Table 3:** Univariate and multivariate logistic regression analysis for grade 4 lymphopenia and grade 3–4 lymphopenia.

Grade 4 Lymphopenia				
Characteristic	Univariate		Multivariate	
	OR (95% CI)	*p* Value	OR (95% CI)	*p* Value
Prior treatment with dimethyl fumarate	5.88 (1.63–21.16).	0.003	7.02 (1.45–34.05)	0.016
Prior treatment with IFNb1a	3.83 (1.06–13.90)	0.031		
CYP2C9 genotype *2*2	26.0 (2.46–314.96)	<0.001	70.88 (4.37–1150)	0.003
Number of prior lines of therapy	1.50 (1.02–2.23)	0.041		
**Grade 3–4 Lymphopenia**				
**Characteristic**	**Univariate**		**Multivariate**	
	**OR (95% CI)**	***p*** **Value**	**OR (95% CI)**	***p*** **Value**
Sex (female)	2.84 (1.13–7.15)	0.023	3.96 (1.28–12.36)	0.017
Age	0.95 (0.91–0.99)	0.021		
CYP2C9 genotype *1*2	4.87 (1.08–7.64)	0.030		
Low lymphocyte value at baseline	1.90 (1.16–3.13)	0.011	1.63 (1.09–2.95)	0.014
Prior treatment with dimethyl fumarate	5.49 (1.55–19.47)	0.004	6.55 (1.28–12.36)	0.017
Prior treatment with IFNb1a	2.50 (1.09–5.71)	0.027		

Abbreviations: OR = odds ratio; CI = confidence interval; IFN = interferon beta 1a. The univariate analysis included all baseline demographic and clinical characteristics; only those with statistically significant results in the univariate analysis are included in this table.

**Table 4:** Safety data.

N = 129	Patients, N (%)
Adverse events (AEs)	60 (46.5)
Serious adverse events	8 (6.2)
Lymphopenia	7 (5.4)
Hepatotoxicity	1 (0.77)
Infectious AEs *	16 (12.4)
COVID pneumonia	7 (5.4)
Conjunctivitis	2 (1.5)
Urinary tract infection	4 (3.1)
Not specified	3 (2.3)
Dose adjustments due to AEs	16 (12.4)
Lymphopenia	15 (11.6)
COVID pneumonia	1 (0.77)

* Two patients had two infectious AEs during follow-up.

## Data Availability

The corresponding authors are willing to provide the data related to this manuscript upon reasonable request.

## Supplementary Materials

The following supporting information can be downloaded at: https://www.mdpi.com/article/10.3390/jcm12206471/s1, Table S1: Continuous and categorical variables evaluated; Table S2: Univariate and multivariate logistic regression analysis for grade 3–4 lymphopenia and grade 4 lymphopenia.

## Appendix A

Realhes Study Group: Aránzazu Sancho-López, Belén Ruiz-Antorán, Teresa Iglesias Hernangómez, Almudena Ramírez-García, Irene Gomez Estévez, Judith Sanabria-Cabrera, Roser Llop Rius, Consuelo Pedrós, Diana Campodonico, Silvia Jiménez-Jorge, Amelia García Luque, Lucienne Costa Frossad França, Eva Montané, Ana Aldea-Perona, Nieves Téllez Lara, Montserrat Bosch Ferrer, Consuelo Rodriguez Jiménez, Elvira Bonilla-Toyos, Julia Sabín Muñoz, Cristina Avendaño-Sola, Maria Rosario Blasco Quilez, Leonor Laredo Velasco, Lourdes Cabrera García, Celia Oreja-Guevara, Concepción Payares Herrera, Gustavo Adolfo Centeno Soto, Patricia Urbaneja Romero, Adrián Roldan, Ana Alonso Torres, Miriam Muñoz Bolaño, Lucía Romero-Pinel, Dolores Rodríguez Cumplido, María Carcelén-Gadea, Javier López Arqueros, Virginia Meca-Lallana, Gina Mejía-Abril, Carolina Díaz-Pérez, Virginia Delgado Gil, María Isabel Lucena, Maria del Rosario Mora Santiago, María Ángeles Lobo-Acosta, Rosso-Fernández CM, Ruth María Aparicio Hernández, Manuel Ángel Silva Cuevas, Mª ángeles Gálvez Múgica, Mónica Aguilar Jiménez, Cristina Ramo-Tello, Melani Núñez-Montero, Marta de Antonio Cusco, Olivia Fernández Quirante, Patricia Mulero Carrillo, Antonia Agustí Escasany, Angela Vidal-Jordana.
