# Supplementary material for: The Need for the Closer Monitoring of Novel Drugs in MS: A Siponimod Retrospective Cohort Study (Realhes Study)
Source: J Clin Med. 2023 Oct 11;12(20):6471. doi: 10.3390/jcm12206471 (PMC10607533; doi:10.3390/jcm12206471)
Supplement: Supplementary file 1 [file jcm-12-06471-s001.zip › jcm-2626131-supplementary.pdf]

The need for the closer monitoring of novel drugs in MS: a Siponimod retrospective cohort study (Realhes Study)

Table of contents

	Page
Table S1. Continuous and categorical variables evaluated	2
Table S2. Univariate and multivariate logistic regression analysis for grade 3-4 lymphopenia and grade 4 lymphopenia.	4

Table S1. Continuous and categorical variables evaluated

Continuous variables	Categorical variables
• Age at diagnosis<br>• Age at start of siponimod<br>• Body mass index<br>• Basal leukocytes<br>• Basal lymphocytes<br>• Number of prior lines of therapy<br>• Initial siponimod dose<br>• Time (months) from last treatment to start of siponimod	• Sex (male/female)<br>• Comorbidities<br>o Hypertension (yes/no)<br>o Autoimmune disorders (yes/no)<br>o Cardiovascular disease (yes/no)<br>o Respiratory disease (yes/no)<br>o Liver disease (yes/no)<br>o Renal impairment (yes/no)<br>• CYP2C9 genotype<br>o *1/*1 (yes/no)<br>o *1/*2 (yes/no)<br>o *2/*2 (yes/no)<br>o *1/*3 (yes/no)<br>o *2/*3 (yes/no)<br>o *3/*3 (yes/no)<br>o Other (yes/no)<br>• Previous treatments received in relation to MS<br>o Interferon beta 1a (yes/no)<br>o Interferon beta 1b (yes/no)<br>o Glatiramer acetate (yes/no)<br>o Fingolimod (yes/no)<br>o Ocrelizumab (yes/no)<br>o Rituximab (yes/no)<br>o Alemtuzumab (yes/no)<br>o Mitoxantrone (yes/no)<br>o Ofatumumab (yes/no)<br>o Ozanimod (yes/no)<br>o Posenimod (yes/no)<br>o Natalizumab (yes/no)<br>o Cladribine (yes/no)<br>o Teriflunomide (yes/no)<br>o Fumaratodimethyl (yes/no)<br>• Concomitant treatments

	◦ Inhibitors of siponimod-metabolizing cytochromes (CYP2C9/CYP3A4) (yes/no)<br>◦ Inducers of siponimod-metabolizing cytochromes (CYP2C9/CYP3A4) (yes/no)<br>◦ Antineoplastics (yes/no)<br>◦ Immunomodulators (yes/no)<br>◦ Immunosuppressants, including corticosteroids (yes/no)<br>• Naive patients (yes/no)<br>• Type of last treatment received prior to siponimod (first-line (Ref)/second-line/inductor)<br>• Adequate washing period (yes/no)<br>• Type of last treatment received prior to siponimod<br>◦ Interferon beta 1a (yes/no)<br>◦ Interferon beta 1b (yes/no)<br>◦ Glatiramer acetate (yes/no)<br>◦ Fingolimod (yes/no)<br>◦ Ocrelizumab (yes/no)<br>◦ Rituximab (yes/no)<br>◦ Alemtuzumab (yes/no)<br>◦ Mitoxantrone (yes/no)<br>◦ Ofatumumab (yes/no)<br>◦ Ozanimod (yes/no)<br>◦ Posenimod (yes/no)<br>◦ Natalizumab (yes/no)<br>◦ Cladribine (yes/no)<br>◦ Teriflunomide (yes/no)<br>◦ Fumaratodimethyl (yes/no)

Table S2. Univariate and multivariate logistic regression analysis for grade 3-4 lymphopenia and grade 4 lymphopenia.

	Grade 3-4 lymphopenia	
	Univariate OR (IC 95%); p	Multivariate OR (IC 95%); p
**Sex**	2.836 (1.126-7.148); 0.023	3.96 (1.28-12.36); 0.017
**Age at diagnosis**	0.95 (0.91-0.997); 0.021	0.94 (0.89-1.020); 0.090
**Age at start of siponimod**	0.92 (0.89-0.998); 0.048	0.91 (0.76-1.101); 0.118
**Body mass index**	0.370	
**Comorbidities**		
Hypertension	0.651 (0.237-1.785); 0.402	
Liver disease	ND	
Renal impairment	ND	
Autoimmune disorders	2.676 (0.553-12.951); 0.206	
Cardiovascular disease	0.081 (0.009-0.698); 0.004	0.076 (0.005-1.125); 0.076
Respiratory disease	ND	
**Basal CBCs**		
Leukocytes x10^3/microL	0.075	
Lymphocytes x10^3/microL	1.90 (1.16-3.13); 0.011	1.63 (1.09-2.95); 0.014
**CYP2C9 genotype**		
*1/*1	1.055 (0.512-2.174); 0.884	
*1/*2	4.874 (1.081-7.640); 0.030	2.539 (0.565-11.414); 0.224
*2/*2	1.111 (0.098-12.595); 0.932	
*1/*3	ND	
*2/*3	ND	
*3/*3	ND	
Other	ND	
Not available	0.281 (0.095-0.832); 0.017	0.293 (0.007-1.928); 0.089
**Prior treatment**		
Interferon beta 1a	2.498 (1.094-5.706); 0.027	1.424 (0.448-4.528); 0.549
Interferon beta 1b	0.982 (0.395-2.439); 0.968	
Glatiramer acetate	0.541 (0.248-1.184); 0.122	
Fingolimod	0.419 (0.181-0974); 0.040	0.277 (0.065-1.1774); 0.08
Ocrelizumab	1.117 (0.266-4.692); 0.880	
Rituximab	1.253 (0.407-3.857); 0.694	
Alemtuzumab	0.543 (0.074-3.990); 0.543	

Mitoxantrone	ND	
Ofatumumab	ND	
Ozanimod	ND	
Posenimod	ND	
Natalizumab	0.316 (0.130-0.765); 0.009	0.173 (0.046-1.291); 0.09
Cladribine	0.549 (0.034-8.984); 0.670	
Teriflunomide	0.651 (0.237-1.785); 0.402	
Fumarato dimethyl	5.494 (1.550-19.472); 0.004	6.55 (1.28-12.36); 0.017
**Number of prior lines of therapy**	0.729	
**Initial dose**	0.374	
**Concomitant treatment**		
Inhibitors of siponimod-metabolizing cytochromes (CYP2C9/CYP3A4)	4.145 (0.494-34.789); 0.158	
Inducers of siponimod-metabolizing cytochromes (CYP2C9/CYP3A4)	2.347 (0.477-11.549); 0.282	
Antineoplastics	ND	
Inmunomoduladores	0.628 (0.239-1.650); 0.343	
Immunosuppressants, including corticosteroids	0.646 (0.266-1.569); 0.332	
**Naive patients**	0.763 (0.269-1.162); 0.611	
**Type of last treatment received prior to siponimod**		
First-line (Ref)	-	
Second-line	0.842 (0.377-1.883); 0.676	
Inductor	0.474 (0.87-2.582); 0.474	
**Adequate washing period**	1.345 (0.524-3.452); 0.538	
**Last treatment received prior to siponimod**		
Interferon beta 1a	1.117 (0.266-4.692); 0.880	
Interferon beta 1b	1.114 (0.196-6.327); 0.903	
Glatiramer acetate	0.967 (0.268-3.496); 0.959	
Fingolimod	0.628 (0.239-1.650); 0.343	
Ocrelizumab	1.714 (0.332-8.861); 0.516	
Rituximab	1.253 (0.407-3.857); 0.694	
Alemtuzumab	0.543 (0.074-3.990); 0.543	
Mitoxantrone	ND	
Ofatumumab	ND	
Ozanimod	ND	
Posenimod	ND	
Natalizumab	1.114 (0.196-6.327); 0.903	

Cladribine	ND	
Teriflunomide	0.282 (0.078-1.022); 0.043	0.172 (0.062-1.938); 0.102
Fumarato dimethyl	9.926 (1.266-77.804); 0.009	8.837 (0.892-82.890); 0.092
**Time (months) from last treatment to start of siponimod**	0.244	

	Grade 4 lymphopenia	
	Univariate OR (IC 95%); p	Multivariate OR (IC 95%); p
**Sex**	1.604 (0.439-5.855;.0.472	
**Age at diagnosis**	0.634	
**Age at start of siponimod**	0.703	
**Body mass index**	0.066	
**Comorbidities**		
Hypertension	ND	
Liver disease	ND	
Renal impairment	ND	
Autoimmune disorders	ND	
Cardiovascular disease	ND	
Respiratory disease	ND	
**Basal CBCs**		
Leukocytes x10^3/microL	0.621	
Lymphocytes x10^3/microL	0.329	
**CYP2C9 genotype**		
*1/*1	0.633 (0.176-2.277); 0.480	
*1/*2	1.926 (0.524-7.077).;0.317	
*2/*2	26.000 (2.460-314.965); <0.001	70.88 (4.37-1,150); 0.003
*1/*3	ND	
*2/*3	ND	
*3/*3	ND	
Other	ND	
Not available	ND	
**Prior treatment**		
Interferon beta 1a	3.831 (1.056-13.898); 0.031	3.232 (0.932-15.441); 0.098
Interferon beta 1b	1.636 (0.401-6.672);.0.489	
Glatiramer acetate	0.926 (0.232-3.703); 0.914	
Fingolimod	0.321 (0.039-2.22); 0.266	

Ocrelizumab	1.375 (0.156-12.131); 0.774	
Rituximab	0.687 (0.082-5.754); 0.727	
Alemtuzumab	3.833 (0.364-40.340); 0.231	
Mitoxantrone	ND	
Ofatumumab	ND	
Ozanimod	ND	
Posenimod	ND	
Natalizumab	1.549 (0.351-6.299); 0.538	
Cladribine	ND	
Teriflunomide	2.575 (0.614-10.795); 0.183	
Fumarato dimethyl	5.880 (1.634-21.160); 0.003	7.021 (1.452-34.051); 0.016
**Number of prior lines of therapy**	1.50 (1.02-2.23); 0.041	1.93 (0.831-4.243); 0.832
**Initial dose**	0,133	
**Concomitant treatment**		
Inhibitors of siponimod-metabolizing cytochromes (CYP2C9/CYP3A4)	1.586 (0.177-14.210); 0.678	
Inducers of siponimod-metabolizing cytochromes (CYP2C9/CYP3A4)	1.211 (0.139-10.555); 0.862	
Antineoplastics	ND	
Inmunomoduladores	2.228 (0.537-9.224); 0.259	
Immunosuppressants, including corticosteroids	0.918 (0.186-4.539); 0.916	
**Naive patients**	ND	
**Type of last treatment received prior to siponimod**		
First-line (Ref)		
Second-line	0.725 (0.192-2.732); 0.634	
Inductor	1.667 (0.166-16.757); 0.664	
**Adequate washing period**	2.258 (0.274-18.615); 0.449	
**Last treatment received prior to siponimod**		
Interferon beta 1a	1.375 (0.156-12.131); 0.774	
Interferon beta 1b	2.260 (0.240-21.278); 0.465	
Glatiramer acetate	ND	
Fingolimod	0.521 (0.063-4.313); 0.539	
Ocrelizumab	1.586 (0.177-14.210); 0.678	
Rituximab	0.687 (0.082-5.754); 0.727	
Alemtuzumab	3.833 (0.364-40.340); 0.231	
Mitoxantrone	ND	
Ofatumumab	ND	

Ozanimod	ND	
Posenimod	ND	
Natalizumab	2.260 (0.240-21.278); 0.465	
Cladribine	ND	
Teriflunomide	1.080 (0.125-9.321); 0.944	
Fumarato dimethyl	3.029 (0.713-12.869); 0.118	
**Time (months) from last treatment to start of siponimod**	0.227	

ND: not available, any of the groups n=0.
